# Protective Effect of Hesperetin and Naringenin against Apoptosis in Ischemia/Reperfusion-Induced Retinal Injury in Rats

**DOI:** 10.1155/2014/797824

**Published:** 2014-01-30

**Authors:** Selcuk Kara, Baran Gencer, Turan Karaca, Hasan Ali Tufan, Sedat Arikan, Ismail Ersan, Ihsan Karaboga, Volkan Hanci

**Affiliations:** ^1^Department of Ophthalmology, Canakkale Onsekiz Mart University, Faculty of Medicine, 17060 Canakkale, Turkey; ^2^Department of Histology and Embryology, Trakya University, Faculty of Medicine, 22030 Edirne, Turkey; ^3^Deparment of Anesthesiology, Dokuz Eylül University, Faculty of Medicine, 35100 Izmir, Turkey

## Abstract

*Purpose*. Hesperetin and naringenin are naturally common flavonoids reported to have antioxidative effects. This study was performed to investigate whether either hesperetin or naringenin has a protective effect against apoptosis on retinal ischemia/reperfusion (I/R) injury. *Methods*. Retinal I/R was induced by increasing the intraocular pressure to 150 mmHg for 60 minutes. Thirty-three male Wistar albino rats were randomised into 5 groups named control, I/R + sham, I/R + solvent (DMSO), I/R + hesperetin, and I/R + naringenin. Animals were given either hesperetin, naringenin, or the solvent intraperitoneally immediately following reperfusion. Thickness of retinal layers and retinal cell apoptosis were detected by histological analysis, tunel assay, and immunohistochemistry assay. *Results*. Hesperetin and naringenin attenuated the I/R-induced apoptosis of retinal cells in the inner and outer nuclear cells of the rat retina. Retinal layer thickness of the naringenin treatment group was significantly thicker than that of the hesperetin, sham, and solvent groups (*P* < 0.05). *Conclusions*. Hesperetin and naringenin can prevent harmful effects induced by I/R injury in the rat retina by inhibiting apoptosis of retinal cells, which suggests that those flavanones have a therapeutic potential for the protection of ocular ischemic diseases.

## 1. Introduction

Disruption of the retinal blood supply, called “retinal ischemia,” decreases the delivery of oxygen and essential nutrients to the retina and results in cell death. Restoration of this disrupted retinal circulation is called “reperfusion” and aggravates ischemic injury by several biochemical processes that include oxidative stress, calcium overload caused by glutamate excitotoxicity, inflammation, cellular necrosis, and apoptosis [1]. Many ocular diseases, such as diabetic retinopathy, ischemic optic neuropathy, acute glaucoma, retinal vascular occlusions, and retinopathy of prematurity, are associated with retinal ischemia/reperfusion (I/R) injury [2]. To define and better understand the pathophysiological mechanisms associated with retinal I/R injury, various experimental approaches have been designed using animal models [3, 4]. Simulating transient retinal ischemia by the high intraocular pressure (IOP) model in rats, which is created by transiently elevating the IOP over ocular perfusion pressure, is well recognized and commonly used in experimental studies [2, 5, 6].

Flavonoids are polyphenolic compounds with various pharmacological properties and act as scavengers of free radicals by OH groups in their molecular structure [7, 8]. According to their structural differences, six major classes of flavonoids are present: flavonols, flavones, flavanones, catechins, anthocyanidins, and isoflavones [9]. Hesperetin (3,5,7-trihydroxy-4′-methoxyflavanone) and naringenin (4,5,7-trihydroxyflavanone) are flavanones abundant in citrus fruits, including oranges and grapefruit, as well as tomatoes and cherries [10]. In nature, these flavanones take glycoside form, which enhances intestinal absorption [11]. Several studies have reported that hesperetin and naringenin have anti-inflammatory, antioxidant, anticarcinogenic, and neuroprotective effects [12–15]. Both hesperetin and naringenin are compounds with 3 hydroxyl groups that maintain a greater antioxidant potency and ability to activate cellular antioxidant preventing enzymes than other flavanones [16, 17].

For this study's purposes, it is important to note that the beneficial effect of hesperetin and naringenin on I/R-induced myocardial, cerebral, renal, and pancreatic injury has been demonstrated [18–21]. However, to the best of our knowledge, no study has yet evaluated apoptosis and structural damage on retinal I/R injury. Thus, we aimed to evaluate the possible protective effects of naringenin and hesperetin on retinal I/R injury model in rats.

## 2. Material and Method

### 2.1. Animals

Thirty-three healthy male Wistar albino rats weighing 250–300 g were obtained from Saki Yenilli Animal Laboratory, Ankara, Turkey. The animals were housed for a 12-h light/12-h dark cycle. Animal experiments were conducted in accordance with the Statement on the Use of Animals of the Association for Research in Vision and Ophthalmology. The institutional review board of the University of Canakkale Onsekiz Mart, Turkey, approved the research.

### 2.2. Ischemia and Reperfusion

Rats were anesthetised with intramuscular ketamine chloride (80 mg/kg: Ketalar, Eczacıbaşı, Istanbul, Turkey) and xylazine (4 mg/kg: Rompun, Bayer, Istanbul, Turkey). Corneal analgesia was achieved by using a topical application of 0.5% proparacaine hydrochloride (0.5% Alcaine; Alcon, USA). To preserve body temperature of the rats, they were placed in containers to cover their body. The anterior chamber of the right eye was cannulated with a 30-gauge infusion needle connected to a saline bottle. Retinal acute ischemia was induced by elevating the bottle to a height of 2 m to maintain an intraocular pressure of 150 mmHg for 60 minutes. Retinal ischemia was confirmed by the loss of red reflex and its return just after reperfusion. After a 60-minute period of ischemia, the IOP was returned to its normal values by removing the infusion cannula. Ofloxacin (0.3%) was applied topically to the eye to prevent infection. The rats in the control group were administered by cannulating a 30-gauge needle into the anterior chamber without elevating IOP.

### 2.3. Drug Administration

Hesperetin (20 mg/kg, Sigma-Aldrich Chemical Co., United Kingdom) and naringenin (20 mg/kg, Sigma-Aldrich Chemical Co., United Kingdom) were freshly prepared by dissolving the powder in dimethyl sulfoxide (DMSO) for intraperitoneal administration. The rats were randomly assigned to five groups: control (*n* = 6), I/R + sham (*n* = 6), I/R + solvent (DMSO) (*n* = 7), I/R + hesperetin (*n* = 7), and I/R + naringenin (*n* = 7). Animals were given either drug (hesperetin or naringenin) or DMSO intraperitoneally immediately following reperfusion.

### 2.4. Tissue Collection and Histological Analysis

The rats were anesthetised by intraperitoneal administration of 80 mg/kg ketamine and 4 mg/kg xylazine, 48 hours after retinal I/R injury. Eyes tissue samples were obtained for histopathological and immunochemistry investigation. Eyes samples fixed with 10% formaldehyde solution for 48 hours, embedded in paraffin. Sections 5-*μ*m thick were obtained using a microtome (Leica, RM2245) and stained with haematoxylin and eosin (H&E). Retinal thickness was quantified in three separate positions: central (100–150 *μ*m from the optic nerve), peripheral (100–150 *μ*m from the ora serrata), and midperipheral (halfway between the central and peripheral). Two representative sections were selected from the same three positions randomly for each eye, from which measurements were taken and their values averaged [22].

### 2.5. Immunohistochemistry in the Retina

The eye samples were fixed in 10% formaldehyde solution and embedded in paraffin. Immunohistochemical reactions were performed according to the avidin biotin-peroxidase complex technique described by Hsu et al. [23]. Five *μ*m-thick sections were obtained, and the slides were air-dried and the tissue deparaffinised. Slides were washed in 0.01 mol/L phosphate-buffered saline (PBS). After washes with PBS, an antigen retrieval solution (0.01 M citrate buffer, pH 6.0) was applied for 10 minutes at 100°C in a microwave oven, and endogenous peroxidase was eliminated by incubation in 3% H_2_O_2_ in pH 7.4 in PBS (0.01 M) for 10 minutes. After washing, specimens were treated with a blocking serum (Labvision, TR-060-UB) at room temperature for 10 minutes. The sections were incubated with rabbit polyclonal anti-Caspase 3 (Abcam, ab4051; dilution 1 : 100) and reacted with tissue specimens at room temperature for 90 minutes. Sections were then washed three times with PBS and incubated with biotinylated secondary antibody (Ultra Vision Detection System-HRP kit, Thermo, Fremont, California, USA). Streptavidin peroxidase (Ultra Vision Detection System-HRP kit, Lab Vision, Fremont, California, USA) was given at room temperature for 20 minutes. 3,3′-Diaminobenzidine (DAB) was used as a chromogen, and sections were counterstained with haematoxylin.

### 2.6. Tunel Assay

Apoptotic cells were visualized by using a terminal deoxynucleotidyl transferase dUTP nick-end labeling (tunel) assay kit (TdT-Fragel TM DNA Fragmentation Detection Kit, Cat. no. QIA33, Calbiochem, USA). In brief, paraffin sections were deparaffinised in the xylene for 3 × 5 minutes, washing with absolute ethanol (2 × 5 minutes), washing once with 95% ethanol and once with 70% ethanol, and after that washing by applying 20 mg/mL proteinase K (20 min.). Endogenous peroxidase activity was inhibited by incubation with 3% hydrogen peroxide. Sections were incubated with an equilibration buffer for 10 to 30 minutes and then with TdT-enzyme, in a humidified atmosphere at 37°C, for 60 minutes. They were subsequently put into prewarmed working strength stop/wash buffer at room temperature for 10 minutes and incubated with a blocking buffer for 30 minutes. Each step was separated by thorough washes in tris-buffered saline (TBS). Labeling was revealed using DAB, counter staining was performed using haematoxylin, and sections were dehydrated, cleared, and mounted [24].

### 2.7. Statistical Analysis

The data were expressed as mean ± standard deviation (SE). Differences among the groups were evaluated using one-way analysis of variance (ANOVA). The Bartlett test was used to determine whether the data were heterogeneous or homogeneous. The Bonferroni multiple comparison procedure was then applied to identify differences between means. Differences were considered significant at *P* < 0.05.

## 3. Results

**Figure 1:**
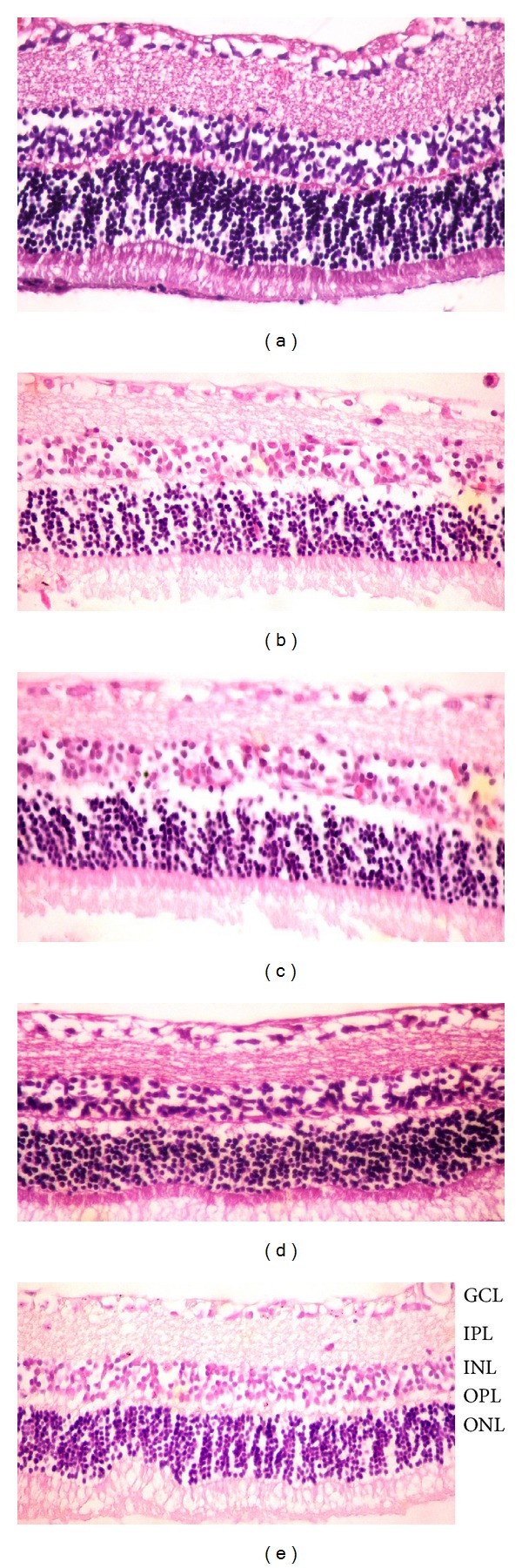
(a) In controls, normal retina architecture was seen; (b) after ischemic injury, severe retinal damage was noted; (c) ischemic injury plus DMSO group, observed reduction in retinal thickness and injuries; (d and e) there was an improvement in the retinal structure in hesperetin-treated and naringenin-treated ischemic rats, respectively. Retina thickness had increased significantly after in the hesperetin- and naringenin-treated compared to ischemic rats. Haematoxylin and eosin staining (×400).

Excepting the naringenin treatment group, 48 hours after retinal I/R injury the overall retinal thickness and other layers of the retina were significantly decreased compared to the control group (*P* < 0.05, Table 1, Figures 1(a) and 1(b)). It is shown that hesperetin and naringenin treatment groups have thicker inner retinal layers when compared to sham and solvent groups (*P* < 0.05, Table 1, Figures 1(b), 1(c), 1(d), and 1(e)). It is also shown that the retinal thickness of the naringenin treatment group was significantly more improved than the hesperetin group (*P* < 0.05, Table 1). In the naringenin treatment group, there was not statistically significant difference in the retinal thickness when compared with the control group.

**Figure 2:**
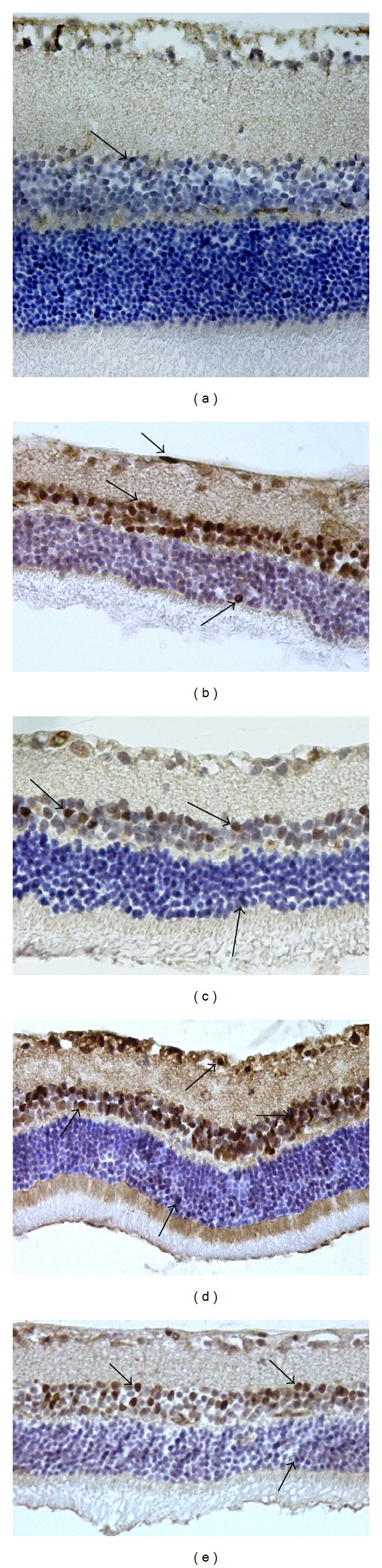
Caspase 3. (a) Control group, a few caspase 3-positive cells; (b) ischemic group; (c) ischemic injury plus DMSO group; (d) ischemia plus hesperetin-treated group, and (e) ischemia plus naringenin-treated group. At the end of the experiment, fewer caspase 3-positive cells were noted in the hesperetin- and naringenin-treated groups than those in the ischemic retinal cells. Arrows: Caspase 3-positive cells. Immunoperoxidase, hematoxylin counterstain (×400).

**Figure 3:**
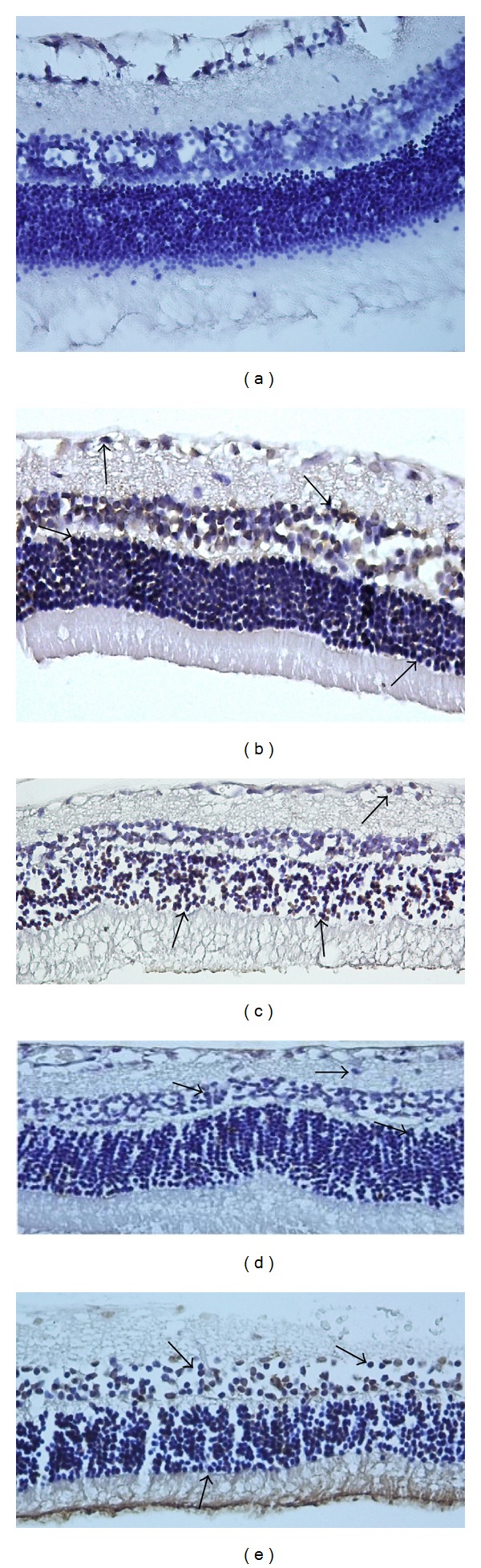
Tunel staining. Representative photographs of tunel staining in control (a), ischemic (b), ischemic injury plus DMSO group (c), ischemic injury treated with hesperetin group (d), and ischemic injury treated with naringenin group in rat retina (e). Positive cells of tunel staining were increased significantly in ischemic rats. However, treatment with hesperetin and naringenin markedly reduced the number of retinal cell apoptosis, respectively. Arrows: tunel-positive cells (×400).

The effects of I/R on retinal cell death were examined by measuring caspase-3 level and DNA fragmentation in nucleus. Tunel staining was used to detect DNA fragmentation of cells undergoing apoptosis. In the control group, the retina cell nuclei were almost negative for tunel staining (Table 2, Figure 2(a)). Significantly more tunel-positive cells were found within the inner nuclear cells in the sham and solvent groups than in the control group (*P* < 0.001, Table 2, Figures 2(b) and 2(c)). By contrast, in rats subjected to I/R with hesperetin or naringenin treatment, significantly less tunel-positive cells were found compared to the other groups (*P* < 0.005, Table 2, Figures 2(d) and 2(e)). It was also demonstrated that the naringenin treatment group has fewer tunel-positive cells than the hesperetin group (*P* < 0.05). We performed immunohistochemistry to detect activated caspase-3. The number of caspase-3 positive cells was significantly less in the retina of rats treated with hesperetin or naringenin than in the retina of the sham and solvent groups, which had retinal damage induced by I/R (*P* < 0.001, Table 3, Figure 3).

## 4. Discussion

This study evaluated the probable protective effects of the two flavanones (hesperetin and naringenin) on the retinal damage caused by I/R in a rat retinal model by studying retinal morphology and immunohistochemistry. The retinal thickness and the quantity of apoptotic cells were compared between I/R groups and the control group. It was found that naringenin and hesperetin markedly reduced retinal cell injury following I/R in comparison to the sham and solvent groups. In addition, we also found that the treatment of naringenin inhibited the apoptosis of the retinal cells and reduced the thinning of the retinal thickness more effectively when compared to hesperetin.

Flavonoids and diets rich with flavonoids have been reported to maintain treatment for several diseases with their powerful antioxidant activity, which also modulates the enzymatic activity [25, 26]. Antioxidant activity of the flavonoids favors by the presence of a 3-hydroxyl group in the heterocyclic ring and a catechol group in ring B, which are also the structural features of hesperetin and naringenin [16, 27]. Although this study implemented a treatment method of intraperitoneal injection of flavonoids, their intestinal absorbable forms include a 2-hydroxyl group (hesperidin and naringin), which appears in citrus fruits naturally and are converted to biologically active forms (hesperetin and naringenin) in vivo [16, 28]. As those have 3-hydroxyl group, hesperetin and naringenin have been suggested to be more effective antioxidants than ascorbic acid and *α*-tocopherol, particularly for urgent antioxidative treatment needs [29]. Flavonoids also have potency to increase ocular blood flow in relation to the quantity of OH groups. Xu et al. [30] showed that intraperitoneal injection of hesperetin and naringenin increased ocular blood flow; thus, the retinal function recovery was facilitated during retinal I/R insult. It was suggested that flavonoids improve endothelium-dependent (enhance NO bioavailability) and endothelium-independent (inhibit the responses to Ca^2+^) vasodilatation after I/R, as well as protect vascular function [29]. Our findings are demonstrating the prevention of structural damage and apoptosis in a retinal I/R injury animal model with the use of naringenin and hesperetin treatments that are compatible with the prior studies.

The neuroprotective effect of flavanones on cerebral I/R injury was shown in recent studies [12, 31–33]. Shi et al. [31] demonstrated that pinocembrin (5,7-dihydroxy flavanone) significantly reduced neuronal loss and brain edema at 24 h after global cerebral I/R with a broad therapeutic time window (intravenously 30 min before ischemia and 30 min, 2 h, and 6 h after reperfusion). Flavonoids seem to sufficiently affect the ischemic site of the neuronal cells when the drug is applied just after I/R. We also aimed to investigate the therapeutic potential of hesperetin and naringenin after I/R because occlusive retinal diseases are nonpredictable. Hesperetin and naringenin were reported to exhibit protective effects in oxidative stress associated neurodegeneration [12, 32, 33]. In addition, Hwang et al. [32] revealed a novel mechanism that hesperetin triggers estrogen receptor (ER)- and TrkA-mediated parallel pathways which induces neuroprotective effect.

This study shows that retinal cell injury seems to occur 2 days after I/R, as demonstrated with the sham group [34]. It is commonly accepted that I/R is associated with increased oxygen-radical production; other factors including excitatory amino acids, and reduced vasodilator reserve leading to retinal cell damage [35–37]. Earlier studies have shown that apoptosis and necrosis are parts of I/R-induced retinal cell damage [1, 38]. There are various apoptotic proteins in apoptotic cell death, including caspase family members. Caspase-3 particularly is a key enzyme required for execution of apoptosis [39]. In the present study, we have seen an increased amount of cells with caspase-3 compared to normal retina. Treatment with hesperetin or naringenin similarly reduced caspase-3 positive cells as compared to the sham and solvent groups. In our tunel study, a significant number of tunel-positive cells were found after I/R. However, the affected cell amount markedly decreased with hesperetin and naringenin treatment immediately after the ischemic period. Consistent with Chiou and Xu [16], this study has shown that naringenin is more protective for retinal cells during the reperfusion period. There were less tunel-positive or caspase-3-positive cells in the ONL than the INL. It was suggested that free radicals penetrate slowly to the outer segment of the retina and so photoreceptor cells are affected at last [40].

An important limitation in this study is that only one dose of both hesperetin and naringenin was studied. Therefore, further studies are needed to evaluate the protective effect of these drugs in different doses. In addition, the current study did not examine the mechanism of the neuroprotective effects of hesperetin and naringenin.

In conclusion, this study is important as it is the first to clearly show the protective role of three-hydroxyl-inclusive flavanones (hesperetin and naringenin) during retinal apoptosis and morphology with the retinal I/R injury in rats. Further investigations are necessary to evaluate the therapeutic potential and mechanism of hesperetin and naringenin for the prevention of ocular ischemic diseases.

## Figures and Tables

**Table 1:** Thickness of the rat's retina (*μ*m).

	GCL	IPL	INL	OPL	ONL
Control	21.8 ± 5.2	50.4 ± 6.4	25.2 ± 5.1	12.1 ± 1.8	50 ± 9.8
Sham	13.4 ± 2.6*	20.2 ± 3.9*	15.6 ± 2.5*	8.6 ± 2.0*	40 ± 6.4*
Solvent	11.3 ± 4.1*	21.3 ± 3.5*	16.2 ± 4.2*	8.1 ± 2.1*	42 ± 8.7*
Hesperetin treatment	9.5 ± 3.1**	20.7 ± 5.4*	17.4 ± 3.7*	8.5 ± 1.7*	32 ± 6.3**
Naringenin treatment	12.2 ± 3.1*	25.4 ± 2.7***	24.2 ± 1.8^#^	9.5 ± 1.4***	47.6 ± 3.4^#^

GCL: ganglion cell layer; IPL: inner plexiform layer; INL: inner nuclear layer; OPL: outer plexiform layer; ONL: outer nuclear layer.

*P < 0.05: comparison to control; **P < 0.05: comparison to control, sham, solvent, and naringenin groups; ***P < 0.05: comparison to control, sham, solvent, and hesperetin treatment groups; ^#^ *P* < 0.05: comparison to sham, solvent groups, and hesperetin treatment groups.

**Table 2:** Number of tunel positive cells in the retina.

	Control	Sham	Solvent group	Hesperetin group	Naringenin group
INL	3.2 ± 0.4	465.28 ± 85.7^a^	392 ± 62.1^b^	170.24 ± 42.4^c^	149.12 ± 25.8^c,d^
ONL	0 ± 0	188.16 ± 36.8^a^	153 ± 18.8^b^	77.44 ± 16.7^c^	69.76 ± 12.7^c,d^

INL: inner nuclear layer; ONL: outer nuclear layer.

^a^ *P* < 0.0001: comparison to control; ^b^ *P* < 0.001: comparison to control and sham groups; ^c^ *P* < 0.005: comparison to control, sham, and solvent groups; ^d^ *P* < 0.05: comparison to hesperetin treatment group.

**Table 3:** Number of caspase-3 positive cells in the retina.

	Control	Sham	Solvent group	Hesperetin group	Naringenin group
INL	13.44 ± 4.2	520.32 ± 65.7^a^	491 ± 74.9^a^	385.92 ± 72.6^b^	338.56 ± 32.8^b^
ONL	2.56 ± 0.4	68.48 ± 12.7^a^	46.2 ± 14.2^c^	38.4 ± 8.1^b^	39.56 ± 9.5^b^

INL: inner nuclear layer; ONL: outer nuclear layer.

^a^ *P* < 0.0001: comparison to control; ^b^ *P* < 0.001: comparison to control, sham, and solvent groups; ^c^ *P* < 0.001: comparison to control and sham.
